# The Leviathan

**Published:** 1841-08

**Authors:** 


### THE WESTERN JOURNAL

Vol. IV.—No. II.

LOUISVILLE, AUGUST 1, 1841.

### THE LEVIATHAN DISCOVERED AT LAST.

During the past year, Albert Koch, a German emigrant, has been exhibiting in St. Louis, New Orleans, Louisville, and Cincinnati, an assemblage of large fossil bones, designed to represent the skeleton of an immense, extinct, amphibious animal; which he has ascertained to be the Leviathan of the Bible, and to constitute a new genus, distinct from the elephant, mastodon, ichthyosaurus, megalonyx, whale, behemoth, and all other existing or extinct monsters, either of land or sea. This happy discovery was made in the diluvial grounds of *La Pomme de Terre,* a branch of the Osage river, in the state of Missouri, lat. 40° and lon. 18° west from Washington.

According to the discoverer, the Missouri Leviathan, or Missourium, as he likewise calls it, had not only a magnitude but peculiarities, which constituted it one of the most remarkable productions of nature. Its length was 32 feet, height 15, breadth through the breast 10, and the tips of its tusks were 20 feet apart; it had no proboscis; but to make up for this deficiency, was furnished with a pair of collar bones, and was web footed! An animal of such size and singularities, could not fail to attract us as well as all others who delight in the wonderful, and we accordingly crowded in with the crowd. Great, however, was our disappointment, in finding nothing more nor less, than a grotesque assemblage, of what in the west are called mammoth bones—in other words, a very good collection of materials for reproducing a skeleton of the gigantic mastodon (*M. Giganteum of Cuvier.*) This fact, sufficiently apparent from a first view of the monstrous superstructure, was rendered absolutely certain, by the inspection of particular bones; such for example as the teeth and tusks, the jaws, the vertebræ and the feet. But, how, it may be asked, could the Leviathan of antiquity be reconstructed out of a mastodon? We answer as follows.

The spine of the mastodon consists of 29 or 30 vertebraæ or joints; but Mr. Koch has strung on an iron wire, no less than 41, drawn from different animals, and placed them at such distances from each other, that their oblique processes do not touch; filling up the intervals with single or double blocks of wood, about two inches thick. In this way he added about 10 feet to the length of the spine, the middle of which he bent upwards: and then suspended the whole (at a suitable height to make a monster) from a strong wooden frame. He next collected 48 ribs, instead of 46, and disregarding their sockets, set their ends along the spine, mostly above the oblique and transverse processes, not neglecting to give them an upward flare. These ribs are not of greater length than those of the mastodon and do not differ from them in form. He then, building downwards, constructed a pelvis, which appears to be composed of the bones of two individuals. He did not, however, apply the articulating surfaces of the ossa ilii to those of the sacrum; but sunk them almost below that bone. Then came the thigh bones, with their heads several inches below the sockets; to which succeeded the bones of the legs, at the same respectful distance, and with their lower ends separated quite as far from the feet. In reproducing and applying the fore-legs, the same rule was followed, by which the scapulæ, instead of embracing the spine, lap over the lower ends of the ribs but a few inches. Thus it was, that he filled up the space between the suspended spine, and the platforms which support the feet. Now for the width, 10 feet from one shoulder joint to the other. This was easily obtained, by causing the ribs to project, laterally, and then not even bringing the scapula into contact with them.

As to the alledged clavicles, we saw two bones suspended by wires in a downward direction, in front of the shoulder blades, but so far separated from each other, as to have required a sternum five or six feet in width! No sternum, however, was found with the other bones. From their height, we could not make a close inspection of these bones, but from their size and appearance, suppose them to have been the two first ribs, of the mastodon, or some other animal, possibly the megatherium, to which they bear a resemblance. At first view, we thought they might even be the recurrent scapulary processes, as those projections had been broken off. Without, however, insisting on any of these conjectures, and especially the last, we may affirm that they are not what they are here made to appear, as no animal having the kind of bones which make up this *compilation*, ever had a clavicle.

Let us descend to the feet and toes. These are precisely those of the mastodon. But the bones are not placed compactly, with their articulating surfaces in contact, but sprawled out to create interspaces, for those webs, which the discoverer coolly informs us, connected the toes together. In the fore-feet one of these toes is fastened to the lower end of the radius instead of the carpus!

We come, finally, to the head, the superior portions of which have decayed. The grinders of the upper jaw have their processes or denticules worn down, while those of the lower are entire, indicating, apparently, that they had belonged to different individuals. Over the mental foramen on the left side of the lower jaw, there is a rough amorphous, bony process, on which the discoverer lays great stress; but, as it is wanting on the other side, and manifestly never existed there, we are constrained to regard it as an exostosis. The tusks are not in their sockets, which are much decayed; but have their maxillary extremities placed near the apertures, and thence pass off, almost horizontally, with their convexities directed forward. The reason assigned for this disposition is, that they had it when discovered. But even admitting the fact, it does not prove that they had that direction during life. As their sockets and ends were both considerably decayed, they were not firmly infixed, and by a mere sinking of the upper jaw, from the superincumbent pressure, would appear to pass off in a horizontal course. In another jaw-specimen of the same kind of animal, exhibited by Mr. K., the tusk advances obliquely downwards, and forwards, having its point turned a little outwards, and upwards, as in the elephant. This was no doubt the position of the tusks in the Missourium.

The assertion that this animal had no trunk or proboscis, seems to be entirely gratuitous, and contrary to all analogy. From the height which it really had, to say nothing of that claimed for it by the discoverer, it was quite impossible that its lips could reach the ground.

We have explained the mode, in which Mr. K. has contrived to give his skeleton dimensions, which it had not in nature. But to show, in a different way, that its size is greatly exaggerated, we shall present a tabular view of some of its principal bones, and of the corresponding parts of Peale's skeleton, in the Philadelphia Museum.

We adopt the admeasurements of Mr. K. as stated in his pamphlet without vouching for their accuracy—the others are from the second volume of Godman's American Natural History.

| BONES. | Koch's Skeleton. | | Peale's Skeleton. | |
|---|---|---|---|---|
| | *Feet* | *Inches.* | *Feet* | *Inches.* |
| Length of tusk, | 10 | 0 | 10 | 7 |
| " " largest grinders, | 0 | 7 | 0 | 7 1-10 |
| Longest vert. including spinous process, | 2 | 6 | 2 | 3 |
| Length of scapula, | 3 | 1 | 3 | 1 |
| " " humerus, | 3 | 5 1-2 | 2 | 10 |
| " " ulna, | 2 | 7 1-2 | 2 | 5 1-2 |
| " " radius, | 2 | 3 3-4 | 2 | 4 |
| " " femur, | 4 | 0 1-2 | 3 | 7 |
| " " tibia, | 2 | 4 3-4 | 2 | 0 |
| " " fibula, | 2 | 6 1-2 | 2 | 2 |

From these admeasurements it appears, that the height of Koch s skeleton, from the forefoot to the top of the scapula, is 8 feet 10 inches, that of Peale's 8 feet 3 inches—difference only 7 inches. But we know, that Peale's is not a large skeleton, as larger detached bones than any in his, have been found. Thus in 1802, Dr. William Goforth, of Cincinnati, dug up at Big Bone Lick, a tusk that measured 11 feet 6 inches. We may then positively pronounce, that the Missouri Leviathan, is not only the common mastodon, but a specimen of the *common size*.

This conclusion, so much at variance with the unqualified assertion of the exhibitor, may justify our devoting a paragraph to the theory on which, we presume, he proceeded in building up the huge amorphous fabric, which he is now imposing on the community, as the "greatest monument of antiquity."

It is quite evident, that the task he proposed to himself, was to construct a Leviathan, out of the bones he had found in the banks of the Pomme de Terre and other streams. To do this, as near as possible according to the 41st chap. of the book of Job, he places it in the water, and gives it web feet; then disposes of its tusks horizontally, separating their tips 21 feet asunder, so that it might not traverse the forest; finally, he takes away its proboscis, "so that thou canst not put a hook into his nose," (vers. 2nd,) and, because its presence would ally his monster too closely to the elephant and the mastodon for popular effect.

In his pamphlet Mr. K. endeavors to present himself as a comparative anatomist; and in his explanations to visiters he speaks as flippantly of the extinct races, as if he had been the Cuvier of the age in which they flourished; but in his advertisement we see the showman far ahead of the naturalist, as will appear by the following extract from one of them, with which we close our notice.

"This skeleton is universally acknowledged as the greatest phenomenon ever presented in natural history.

"In viewing it, the observing and thoughtful man will be lost in wonder and astonishment, at the immensity of the vast relic, and how shall we express our conception of this monstrous animal when in the full zenith of his mountains of flesh and rivers of blood, and in all the vipor of Omnipotent life, he moved in awful and terrific grandeur on the face of the earth, the sovereign masterpiece and proudest monument of all creation."

### ELEPHANTS AND MASTODONS.

Having dipped into zoology, so far as to notice an exhibition of huge fossil bones, we are constrained to add a page, on the anatomical characters of the extinct elephants and mastodons, whose remains have been found in various parts of the West. To many of our readers, this may not be acceptable, because they are already acquainted with the subject, but there are others who live in places so remote, that books ef zoological science seldom or never fall into their hands. To such what we propose to say may not be unwelcome.

The elephants and mastodons constitute two distinct, but kindred genera, belonging to the family of *proboscidia*, of the order *Pachydermata*, of Cuvier—the former of these terms indicating the presence of a proboscis or trunk, the latter a covering of thick hide. We have seen both in the elephant, but neither in the mastodon; because of the perishable character of those parts. In ascribing them to the latter, therefore, naturalists have been guided by analogy. Let us consider these genera separately.

The genus ELEPHAS of Linnæus, is characterized by eight grinders, or molar teeth, two in each side of each jaw; by two tusks, projecting from the anterior part of the upper jaw, and by a proboscis. But to make the generic character complete, we must attend to the surfaces of the teeth. These are flat, and instead of having a layer of enamel spread over them, present that substance in transverse plates, rising up through the substance of the tooth, and separated from each other by thicker plates of bony matter. The tusks are solid; and as they descend from the jaw, diverge from each other. Their points turn upwards, and consequently their convexity is directed towards the ground.

Two living species of elephant are known, one of which inhabits Africa, the other India. In addition, the teeth of another species, or perhaps, of more than one, are found both in the old world and the new. To this species, Blumenbach has given the specific name *primogenius*. Its detached grinders have been repeatedly found in the western states, in our alluvial grounds, sometimes tolerably solid, but more commonly in a crumbling condition, the bony part being converted into a friable substance almost as white as chalk. The plates of enamel are in pairs joined together, at the side of the tooth, and exhibit the appearance of the end of an elastic tube, compressed between two planes, until its sides are brought nearly parallel to each other. These grinders are worn down obliquely, so that the distance from the crown to the root, is less at one end than the other. The tusks of this elephant have not been distinguished from those of the mastodon, with the bones of which, the grinders we have described have generally been found. As yet, no skeleton has been discovered, and it has, therefore, been conjectured that the *E. primogenius* became extinct at an earlier period than the mastodon; so early, indeed, that the bones have entirely decayed. Nevertheless, we know still more of the osteology of this animal than of the mastodon, as it is, in fact, no other than the mammoth or arctic elephant found, at the beginning of this century, embedded in ice, near the mouth of the river Lena, in Siberia.

The teeth of the genus Mastodon, differ from those we have just described, first in number, second in the form of their surfaces. They consist of four in each side of both jaws, which, however, are not all present at the same time, as the two anterior are milk teeth, and, falling out, are not replaced. The surfaces of these teeth present from two to five transverse wedge shaped projections, deeply indented in the middle, so as to give the appearance of two ranges of conical or teat-like processes—whence the name of the animal. The enamel of these grinders is not disposed in vertical plates as in the elephant; but spread over the surface, so that when the processes are worn down, each one presents a bony surface, more or less rhomboidal, surrounded with a margin of enamel. The tusks of the mastodon are solid, but generally so far decomposed as to break when removed, except they be carefully handled; their places of insertion in the intermaxillary bone of the upper jaw, and their direction from it, are the same as in the elephant.

Cuvier has recognized several species of this genus; but one of them, however, is well known—the *M. giganteum*, with the remains of which we are familiar in the western country. The others are much smaller, and no skeleton of any one of them has been yet exhumed. The bones of the gigantic mastodon have been found in every quarter of the globe; but more frequently in the valley of the Mississippi than in any other country. The size of this animal seems to have been about the same with that of the Asiatic elephant.

### THE TETRACAULODON.

We shall conclude with a brief notice of the remains of an animal confounded with the mastodon, till twelve or fourteen years ago, when the late lamented Dr. Godman separted it into a new but closely allied genus. The characters of the grinders are so nearly the same with those of the mastodon, that we need not, for our present purpose, state them. The essential difference in the dental system of the two, is to be found in *a pair of short tusks in the chin, or anterior part of the lower jaw*; giving to the animal four instead of two, as in the mastodon, and procuring for it the name prefixed to this article, which signifies four tusks. The specimen from which the Doctor made his description was small, and appeared to be young; but before his paper was read in the philosophical society, he came to the knowledge of another, which must have been nearly or quite as large as the mastodon of Peale's Museum. Since the death of Dr. G. his friend Dr. Hays, has read a paper to the same society, in which he confirms this discovery, and endeavors to show that more than one species existed.

We trust that such of our friends as have the good fortune to meet with lower jaws of what are called mastodons, will examine for these small tusks, or the sockets from which they have fallen out.

